# Chemical Degradation of PSF-PUR Blend Hollow Fiber Membranes—Assessment of Changes in Properties and Morphology after Hydrolysis

**DOI:** 10.3390/membranes11010051

**Published:** 2021-01-12

**Authors:** Wioleta Sikorska, Monika Wasyłeczko, Małgorzata Przytulska, Cezary Wojciechowski, Gabriel Rokicki, Andrzej Chwojnowski

**Affiliations:** 1 Nałęcz Institute of Biocybernetics and Biomedical Engineering, Polish Academy of Sciences, Trojdena 4 Street, 02-109 Warsaw, Poland; mwasyleczko@ibib.waw.pl (M.W.); mprzytulska@ibib.waw.pl (M.P.); cwojciechowski@ibib.waw.pl (C.W.); achwojnowski@ibib.waw.pl (A.C.); 2 Warsaw University of Technology, Noakowskiego 3 Street, 00-644 Warsaw, Poland; gabro@ch.pw.edu.pl

**Keywords:** partly degradable hollow fiber membranes, hydrolysis process, PSF-PUR membranes, PUR degradation

## Abstract

In this study, we focused on obtaining polysulfone-polyurethane (PSF-PUR) blend partly degradable hollow fiber membranes (HFMs) with different compositions while maintaining a constant PSF:PUR = 8:2 weight ratio. It was carried out through hydrolysis, and evaluation of the properties and morphology before and after the hydrolysis process while maintaining a constant cut-off. The obtained membranes were examined for changes in ultrafiltration coefficient (UFC), retention, weight loss, morphology assessment using scanning electron microscopy (SEM) and MeMoExplorer™ Software, as well as using the Fourier-transform infrared spectroscopy (FT-IR) method. The results of the study showed an increase in the UFC value after the hydrolysis process, changes in retention, mass loss, and FT-IR spectra. The evaluation in MeMoExplorer™ Software showed the changes in membranes’ morphology. It was confirmed that polyurethane (PUR) was partially degraded, the percentage of ester bonds has an influence on the degradation process, and PUR can be used as a pore precursor instead of superbly known polymers.

## 1. Introduction

Membranes are widely used in various branches of science and technology. Depending on the properties of membranes, they can be used, among others, to separate molecules of different sizes [1,2,3,4,5,6,7], culture [4,8,9,10,11], or even in a drug delivery system (DDS) [12,13,14]. They are increasingly used in medicine, including tissue engineering, the main objective of which is the regeneration or even transplanting of damaged tissues or organs whose function can hardly be restored by conventional treatments [15]. They can occur in various forms, including as 2D membranes, among other uses for skin regeneration [16], in 3D form as scaffolds as a cell support during cultivation [8,17,18], encapsulation of active substances/cells in DDS [12,13,14], and even in the form of HFMs, for examples in dialysis [6,19] in bioreactors among others for cell culture [4,9,10,11,20]. A feature of the mentioned above membranes is their semipermeable structure.

The production method of HFMs is spinning, which can be divided into four general types. For medical purposes the most common is the wet phase inversion spinning method [4]. In this technique, the polymer membrane solution is extruded directly into a coagulant bath, mainly water with detergent, where phase separation occurs [4,21]. The phase inversion process is also used for the production of 2D membranes, scaffolds, or capsules.

The HFMs are mainly made of synthetic, hydrophobic polymers such as polysulfone (PSF), polypropylene (PP), polyvinylidene fluoride (PVDF), polyethersulfone (PES), polytetrafluoroethylene (PTFE), or cellulose acetate (CA) [4,22,23,24,25]. The degradable polyesters such as poly (lactic acid) (PLA), poly (ε-caprolactone) (PCL), poly (lactic-co-glycolic acid (PLGA), or PUR are also used as a material [4,21,26]. Moreover, the above materials could be used for HFMs alone or in blends. The blending of polymers is a simple method to overcome some limitations of single materials, such as inadequate or no degradation, poor mechanical properties. It is possible to provide also adjustment specific qualities like tunable hydrophilicity, elasticity, and even selective permeability [2,4,21,23,24,26,27,28,29,30,31]. Obtaining material from a few polymers is also possible by copolymerization technique, but it is a more complicated method and requires appropriate laboratory facilities. A cheaper and simpler method than copolymerization is the blending technique. This method is increasingly used in the production of HFMs for bioartificial organs or tissue engineering applications [4,31,32]. For instance, to reduce the stiffness of a degradable material it is possible to blend it with a more elastic polymer, like PCL. Such treatment will reduce degradation rates and will provide better mechanical resistance of the polymeric scaffold [4,33].

The effect of blended HFMs also helps to reduce or eliminate the fouling resistance that occurs by the presence of biological macromolecules that adhere very strongly to hydrophobic surfaces of membranes. This is particularly desirable for membranes in biotechnological applications, especially in contact with proteins and microorganisms [4,31]. For example, obtaining blended HFMs from stable and (bio)degradable polymers affects the gradual removal of decomposition material that increases porosity which in turnaffects permeability and delimits the effects of the fouling process. This occurrence should influence good efficiency of membrane processes even over long periods of usage [21,26,28].

Furthermore, helpful methods to eliminate fouling can be, for example, heat and plasma treatment [4,34,35,36,37], coating or grafting of hydrophilic polymers onto membranes’ surfaces [33,35,38,39,40,41,42,43], or through the addition of hydrophilic pore precursors to a polymer solution or other admixture like nanoparticles [6,19,25,44,45,46,47,48]. The modification could be influenced by an increase of the hydraulic permeability and/or the hydrophilicity of membranes. This will improve membrane compatibility and fouling resistance. Such HFMs could be potentially used in tissue engineering as membrane bioreactors or as scaffolds [4,22,23,40].

The aim of this study was to obtain semipermeable HFMs using a polymer blend of PSF and synthesized PUR and evaluate the possibility of partial degradation of obtained membranes by assessing changes in transport-separation properties and morphology after the hydrolysis process while maintaining a constant membrane cut-off point. Additionally, in some cases, the addition of a pore precursor to membrane forming solution was used. Potentially, partial degradation would extend the useful life of the membranes. Received PURs, in their structure, contain ester bonds that potentially undergo degradation processes. The membranes were received in constant PSF:PUR weight ratio 8:2, however, there were changes in used solution, type of PUR, or PVP addition. The degradation of PUR in HFMs was carried out using the flowing method with 1M NaOH solution. The obtained membranes before and after hydrolysis were characterized using UFC for pure water, retention coefficient for selected markers, the weight loss measurement, and FT-IR spectroscopy. The observation of membranes’ morphology was achieved by using the SEM. Furthermore, the structure of pores was evaluated using the MeMoExplorer™, an advanced software designed for computer analysis of the SEM photomicrographs [26,49,50]. The evaluation of the effect of the pore precursor or the type of the used solvent was compared by the MeMoExplorer™ Software. The results of the obtained blended HFMs before and after hydrolysis were compared and presented in this paper.

## 2. Experimental

### 2.1. Material

#### 2.1.1. PUR Synthesis

Dimethyl carbonate (DMC) from Carl Roth (Karlsruhe, Germany) 1,4-butanediol from Sigma-Aldrich (St. Louis, MS, USA), 1,5-pentanediol from Sigma-Aldrich, titanium (IV) butoxide (Ti(OBu)_4_) from Sigma-Aldrich, dimethyl succinate (BDM) from Carl Roth, isophorone diisocyanate from Sigma-Aldrich, dichloromethane from Sigma-Aldrich, hydrochloric acid (HCl) from Chempur (Plymouth, UK), water 18.2 MΩ from MiliQ installation (DI) were used for PURs synthesis.

#### 2.1.2. HFMs Preparation and Characterization

*N*,*N*-Dimethylformamide (DMF) from Chempur; *N*-methyl-2-pyrrolidone (NMP) from Fluka (Charlotte, NC, USA); polyvinylpyrrolidone (PVP) from Sigma-Aldrich; sodium hydroxide (NaOH) from POCH; water 18.2 MΩcm from MiliQ installation, PSF 1700 NT LCD from Dow Corning, M.W. 70 kD, polyvinylpyrrolidone (PVP) from Sigma-Aldrich, polyethylene glycols (PEG) M.W. 4, 15, and 35 kD from Fluka, chicken egg albumin (CEA) M.W. 45 kD from Sigma-Aldrich, bovine serum albumin (BSA) M.W. 67 kD from Fluka were used for obtaining, hydrolyzing, and characterization of the HFMs.

### 2.2. PUR Synthesis

**Figure 1:**
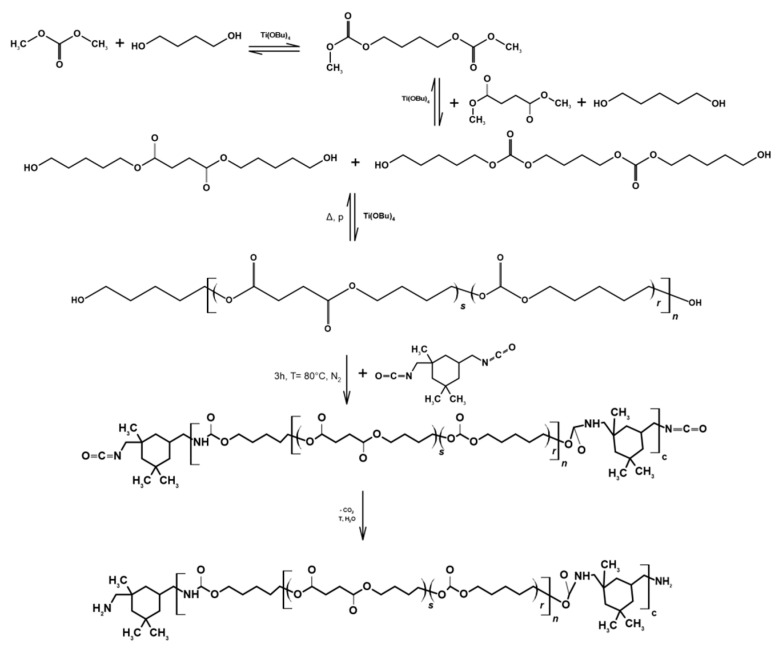
The scheme of the PUR synthesis.

PURs were synthesized using methods presented in [51,52]. Finally, two polymers with different percentages of ester bonds (marked as PUR 1 with ≈80% molar content of ester bonds, and PUR 2 with ≈90% molar content of ester bonds) with the structure presented in Figure 1 were obtained in five steps of the synthesis:

(1) Synthesis of tetramethylene bis(methyl carbonate)
(2) Glycolysis;
(3) Polycondensation;
(4) Reaction with an excess of diisocyanate;
(5) Synthesis of poly(ester-carbonate-urea-urethane).

### 2.3. HFMs Preparation

HFMs were obtained using the dry/wet-spinning, phase inversion technique through extrusion of polymeric solution. PSF* and PUR were mixed with solvent (NMP or DMF) in different flasks and in the next step received solutions were mixed together for 24 h. Finally, eight different HFMs marked as PSF-PUR-1, PSF-PUR-2, PSF-PUR-3, PSF-PUR-4, PSF-PUR-5, PSF-PUR-6, PSF-PUR-7, PSF-PUR-8 (differences in used solvent or PUR) were obtained. Differences between the membranes are presented in Table 1. The PSF:PUR weight ratio was constant: 8:2.

For PSF-PUR-4,5,6,8 PVP was added after complete PSF dissolution.

The membranes and modules were obtained in similar conditions to ensure repeatability of the process (obtaining and modules preparation were presented in previous works: [26,28].

### 2.4. Membranes Hydrolysis

The membranes were treated with a 1M NaOH solution using the flowing method. First, 1 dm^3^ of the NaOH solution was passed through the module from 1 to 3 days. After the hydrolysis process the membranes were treated with demineralized water in order to remove the NaOH solution.

### 2.5. Membranes Characterization

The membranes were characterized twice—before and after the hydrolysis process due to comparison of changes in membranes properties after the hydrolysis process.

#### 2.5.1. Ultrafiltration Coefficient

The ultrafiltration coefficient (UFC) was calculated according to the formula:

(1) $$UFC=\;\frac{v}{p\cdot t\cdot a}$$

where: *v*—the volume of pure water (cm^3^); *p*—transmembrane pressure (hPa); *t*—the time of measure (min); *a*—nominal membrane’s area in a module (m^2^).

The hydraulic permeability was measured as a volume of pure water passed through the membrane’s walls during the period of established time under 200 hPa transmembrane pressure.

#### 2.5.2. Cut-Off Evaluation via Retention Measurements

The membrane retention (%) was defined as:

(2) $$R=\left\{1-\left(\frac{C_{P}}{C_{F}}\right)\right\}\cdot 100\%$$

where: *R*—retention coefficient; *C_P_*—concentration of marker in permeate (g/dm^3^); *C_F_*—concentration of marker in the feed (g/dm^3^).

The concentrations of markers (1 g/dm^3^) were evaluated by a UV-spectrophotometer (HITACHI U-3010, Tokyo, Japan) at 190 nm wavelength for PEGs: 4, 15, and 35 kD and 280 nm for CEA 45 kD and BSA 67 kD.

#### 2.5.3. Mass Measurements

The dry modules of membranes were weighted before and after hydrolysis in order to evaluatechanges in masses to estimate partial PUR degradation.

#### 2.5.4. SEM Analysis

SEM (a Hitachi TM-1000 microscope) was used to characterize morphology of the membranes before and after the hydrolysis process. The membranes were cut in liquid nitrogen for avoiding deformation during fracture and coated with a 10 nm gold layer, using a sputtering device (EMITECH K 550 X, Al Twar, Dubai).

#### 2.5.5. MeMo Explorer™Analysis

The evaluation of SEM images analyzed on the basis of comparison of two images with the human eye is not a precise method, because it is impossible to determine exactly what numerical changes occur in the pore size. In order to more precisely assess morphology changes (in pore size), selected samples were analyzed with the MeMoExplorer™ Software. This program proposes the adoption of images obtained during the SEM analysis of membranes for the analysis of binary time series as well as extension to images of larger dimensions and/or image sequences. In the former paper [53] results of the evaluation of the influence of:

(1) hydrolysis;
(2) different solvents;
(3) pore precursor addition;

On the porosity in various types of membranes, as well as:

(4) porosity parameters’ stability in the series of samples drawn from various membranes were described.

In the experiments, eight types of membranes before hydrolysis (denoted by #1,#2, #3, #4, #5, #6, #7, #8) and eight types after hydrolysis (denoted by #1h, #2h, #3h, #4h, #5h, #6h, #7h, #8h) were taken into consideration.

Besides, the results of evaluation of the above-mentioned factors’ influence on the stability of porosity parameters in eight size-classes of pores will be described.

Size-classes, denoted by ***j***∊{1, 2, 3, 4, 5, 6, 7, 8}, were established according to practical reasons, as described in Table 2.

In each original SEM image of a membrane section about 20 image-segments were selected, all being of the same surface *S*.

Based on the above-mentioned results the following basic statistical parameters were calculated:

- mean values of the pore areas in given classes of membranes obtained by using given technologies, covered by pores of given size-classes:
- standard deviations of the above-mentioned variables.

The results were used for calculation of the following porosity characteristics:

(a) *porosity factors* ($PF_{i,j,k}^{t}$) for given technology (*t*), type of membrane (*i*), and size-class of pores (*j*):
  (3) $$PF_{i,j,k}^{t}=\frac{s_{i,j,k}^{t}}{S}\cdot 100\%;$$
(b) *instability coefficient* ($d_{i,j,k}^{t}$) of general porosity factors, for given technology *t*, type of membrane *i*, and size-class of pores *j*, calculated within the sets of image segments:
  (4) $$d_{i,j,k}^{t}=\frac{Std\left(PF_{i,j,k}^{t}\right)}{Av\left(PF_{i,j,k}^{t}\right)}\cdot 100\%.$$

#### 2.5.6. Fourier-Transform Infrared Spectroscopy Analysis

FT-IR spectra were recorded on a Nicolet iS5 Mid Infrared FT-IR spectrometer equipped with iD7 Attenuated Total Reflectance (ATR) Optical Base.

## 3. Results and Discussion

### 3.1. UFC

UFC was evaluated for the membranes according to Equation (1) in two steps: before (marked as UFC) and after the hydrolysis process (marked as UFC_H_). The values of the UFCs with standard deviations are presented in Table 3.

The increase of the UFC after the hydrolysis process is observed for all of the membranes. The UFC before the hydrolysis increased from 0.0380 ± 0.0030$\frac{{\mathrm{cm}}^{3}}{\min\cdot m^{2}\cdot \mathrm{hPa}}$ for the PSF-PUR-5 membrane to 2.76 ± 0.03$\frac{{\mathrm{cm}}^{3}}{\min\cdot m^{2}\cdot \mathrm{hPa}}$. for the PSF-PUR-3 membrane. After the hydrolysis process the UFC values were in the range from 0.0830 ± 0.0350$\frac{{\mathrm{cm}}^{3}}{\min\cdot m^{2}\cdot \mathrm{hPa}}$. for the PSF-PUR-5 membrane to 26.3 ± 0.2$\frac{{\mathrm{cm}}^{3}}{\min\cdot m^{2}\cdot \mathrm{hPa}}$. for the PSF-PUR-7 membrane. The highest increase between UFC and UFC_H_ is observed for the PSF-PUR-7 membrane (28) and the lower increase between UFC and UFC_H_ for the PSF-PUR-3 membrane (1.6). The PSF-PUR-3 and PSF-PUR-7 membranes were obtained from the same PUR and solvent (NMP), but in PSF-PUR-7 membranes the PVP addition was used.

### 3.2. Molecular Weight Cut-Off Measurements

**Figure 2:**
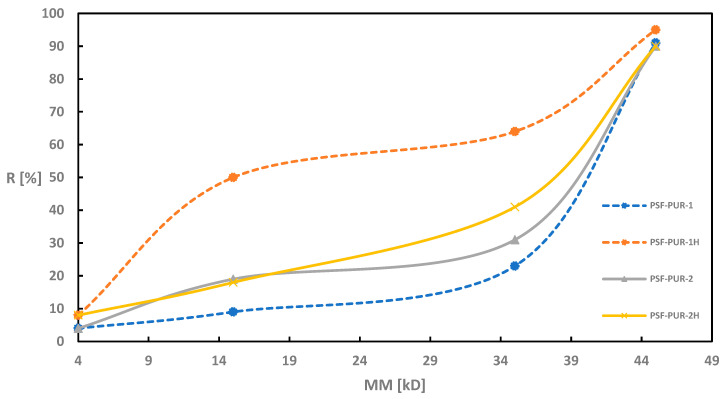
Retention coefficient values for different markers for PSF-PUR-1 and PSF-PUR-2 membranes before (PSF-PUR-1, PSF-PUR-2) and after (PSF-PUR-1H, PSF-PUR-2H) hydrolysis.

**Figure 3:**
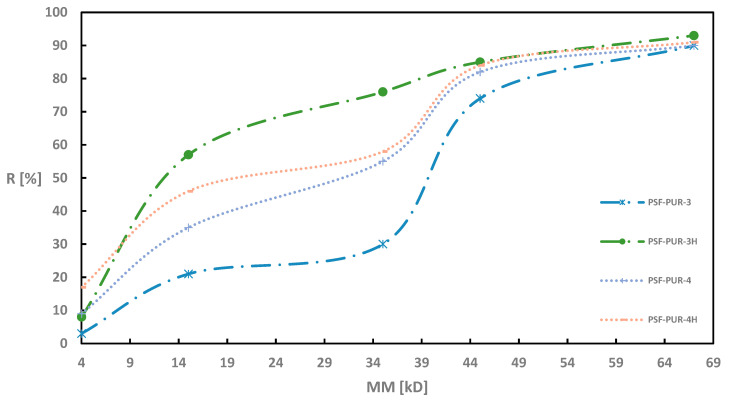
Retention coefficient values for different markers for PSF-PUR-3 and PSF-PUR-4 membranes before (PSF-PUR-3, PSF-PUR-4) and after (PSF-PUR-3H, PSF-PUR-4H) hydrolysis.

**Figure 4:**
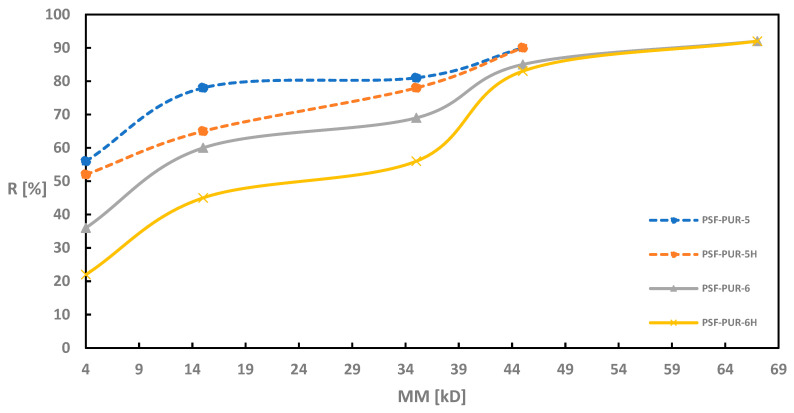
Retention coefficient values for different markers for PSF-PUR-5 and PSF-PUR-6 membranes before (PSF-PUR-5, PSF-PUR-6) and after (PSF-PUR-5H, PSF-PUR-6H) hydrolysis.

**Figure 5:**
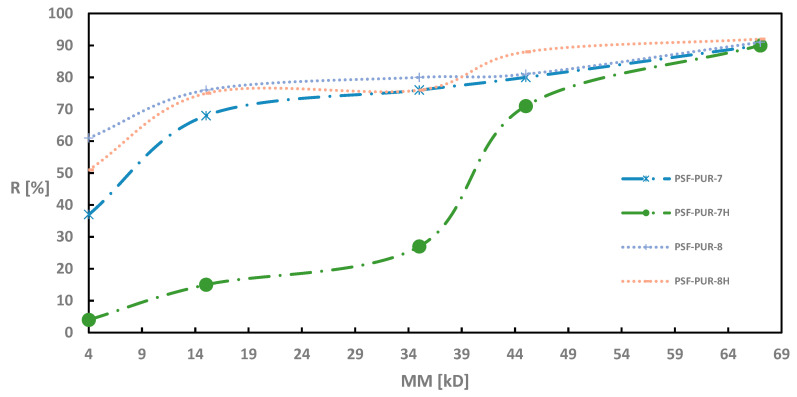
Retention coefficient values for different markers for PSF-PUR-7 and PSF-PUR-8 membranes before (PSF-PUR-7, PSF-PUR-8) and after (PSF-PUR-7H, PSF-PUR-8H) hydrolysis.

As in the case of UFC evaluation, also during retention measurements all modules were tested before and after the hydrolysis process. Figure 2, Figure 3, Figure 4 and Figure 5 of the relationship between the degree of retention and the molar mass of the marker are presented below.

For the membranes without the addition of a PVP (PSF-PUR-1, PSF-PUR-2, PSF-PUR-3 and PSF-PUR-4), the tendency to increase the percentage retention of individual markers after hydrolysis is maintained. The largest differences were obtained for the membranes PSF-PUR-1 and PSF-PUR-3. The smallest changes were obtained for PSF-PUR-4.

On the other hand, membranes obtained with the addition of PVP showed a reverse tendency after hydrolysis. Here, the percentage retention of individual markers was lower after the hydrolysis process (the only exception is the percentage retention for the CEA marker in the PSF-PUR-8 membrane, where a higher result was obtained than before hydrolysis). The dependence of very high retention percentages for PEG 4 is also noticeable.

The degree of marker retention presumably may be affected by the following factors:

(a) percentage of hydrolyzed polymer—in the works cited from the literature, the degradable polymers were almost completely degraded, which was not achieved in this work—the cause may be too small volume of NaOH solution passed through the membranes;

(b) size of pores formed after degradation—the amount of hydrolyzed polymer affects the size of the pores obtained after the hydrolysis process (the greater the loss of polymer, the greater the probability of obtaining larger pores, which allows the passage of compounds with higher molecular weights);

(c) partially degraded polymer may cause the formation of polarized bonds—the ester groups present in the structure of the degradable polymer, activated during the hydrolysis process, may affect formation of hydrogen and ionic bonds between polymer molecules and marker molecules, thus retaining them on the membrane;

(d) the effect of solvation causing the swelling of the polymer—ions formed during the hydrolysis can interact with the molecules of the marker solvent (water), leading to the formation of the so-called solvates which in the next step would cause the PUR to swell and block the pores.

Due to the partial loss of PUR in the hydrolyzed membranes, the most likely scenario is the scenario “c” + “d”, because the partial degradation of PUR could result in the formation of hydrogen bonds between hydrogen atoms from marker molecules and oxygen atoms in the carboxyl groups formed after the degradation of –COOH. The resulting hydrogen or ionic bonds could have a stronger effect on the retention percentage than the size of the pores obtained after the hydrolysis process. On the other hand, the swelling of the polymers due to solvation could effectively block the pores, making the marker molecules less freely permeable through the membrane wall. In the case of PUR swelling, an additional factor influencing this phenomenon is the location of PUR polymer chains in the membrane structure (the more PUR polymer chains at the membrane wall surface, the greater the likelihood of an increased retention factor for markers, especially CEA and BSA, due to their nonlinear structure).

### 3.3. Mass Measurement

Percentage weight loss of PUR after the hydrolysis process is presented in Table 4. Due to the partial removal of PVP, no weight loss measurements were made for PSF-PUR-5, PSF-PUR-6, PSF-PUR-7, PSF-PUR-8 membranes.

The percentage removal of PUR is in the range from 34% for PSF-PUR-2 to 97% for the PSF-PUR-3. The percent of the removal is related to the differences in the retention coefficients: the smallest changes in retention coefficients after the hydrolysis process and PUR removal for PSF-PUR-2 and the largest changes in retention coefficients after the hydrolysis process and PUR removal for the PSF-PUR-3 is observed. It can be observed that PUR-2 is a better hydrolyzing polymer under the same conditions of the hydrolysis process since for both membranes (PSF-PUR-3 and PSF-PUR-4) the percentage of PUR’s mass loss after hydrolysis is greater than for membranes obtained from PUR-1.

### 3.4. Scanning Electron Microscopy Analysis

**Figure 6:**
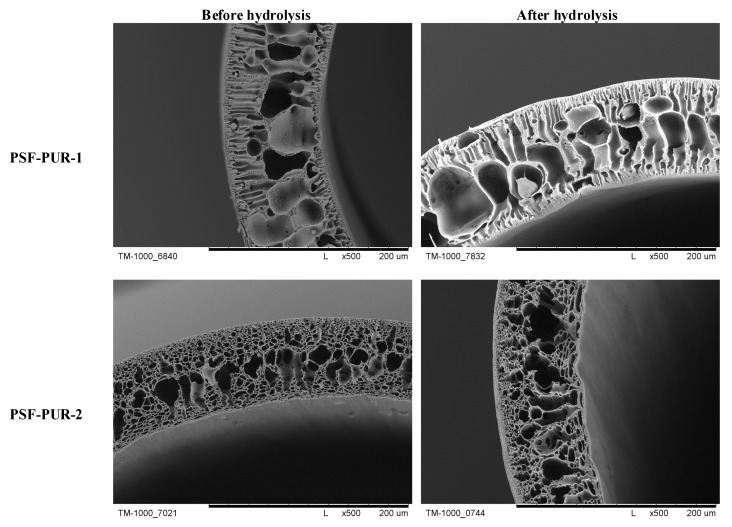
The part of the crosssection of the PSF-PUR-1 and PSF-PUR-2 membranes before and after hydrolysis.

**Figure 7:**
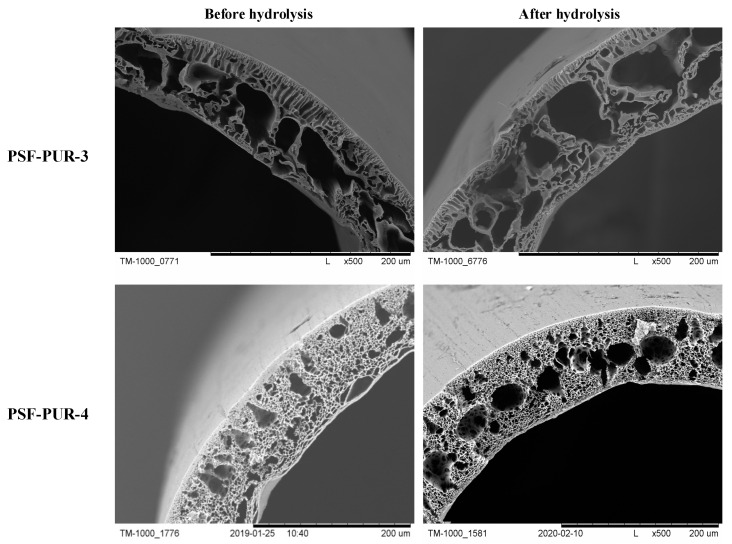
The crosssection and part of the crosssection of the PSF-PUR-3 and PSF-PUR-4 membranes before and after hydrolysis.

**Figure 8:**
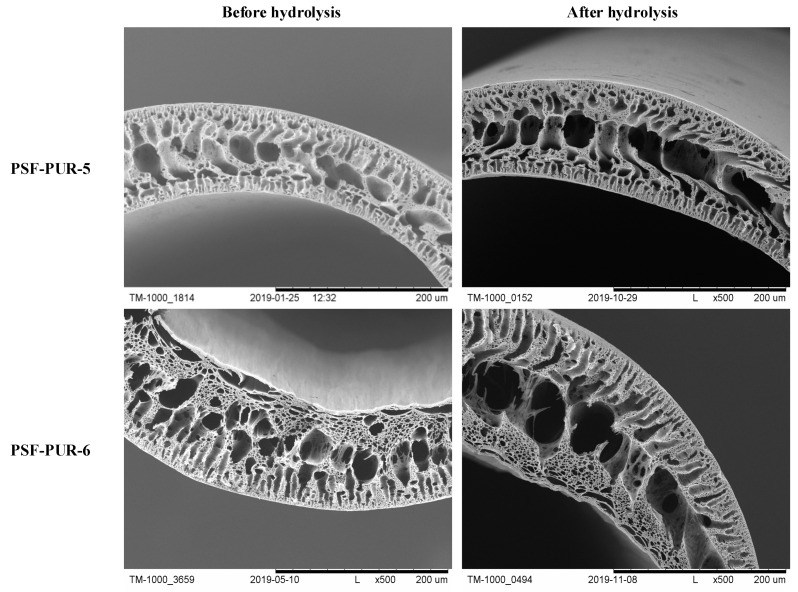
The crosssection and part of the crosssection of the PSF-PUR-5 and PSF-PUR-6 membranes before and after hydrolysis.

**Figure 9:**
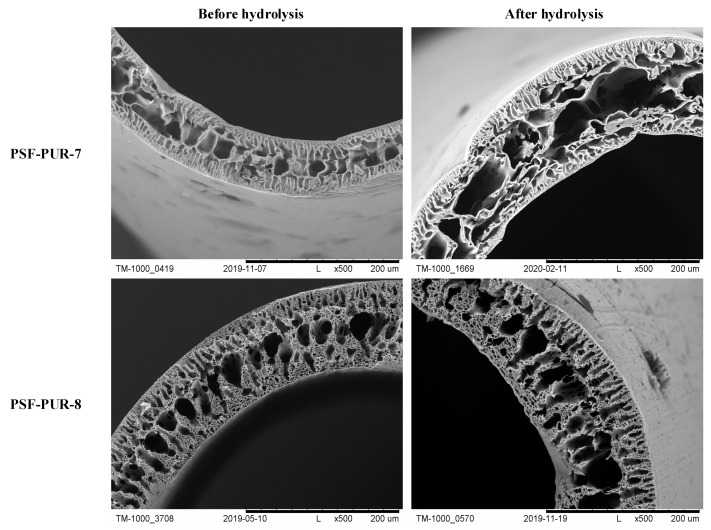
The crosssection and part of the crosssection of the PSF-PUR-7 and PSF-PUR-8 membranes before and after hydrolysis.

The membranes after preparation were subjected to analysis using SEM. Crosssection images and their parts were taken at several magnifications to assess the morphology and changes occurring after the hydrolysis process. The part of crosssections of the membranes before and after the hydrolysis process are presented in Figure 6, Figure 7, Figure 8 and Figure 9 (comparison of cross sections and part of cross sections for all membranes is presented in Supplementary Material–Figures S1–S8).

SEM analysis showed that the obtained membranes have an asymmetric structure, in most cases with a clear epidermal layer. No skin layer is seen for the membranes PSF-PUR-2 and PSF-PUR-4 and it can be caused by using DMF as a solvent. All membranes in the supporting layer had pores with the structure of holes (smaller or larger) maintaining similar morphology among the series of obtained membranes. When assessing the morphology of membranes before and after hydrolysis, a difference in pore size is noticeable. The hydrolysis process influenced their enlargement, also causing the formation of macropores.

### 3.5. MeMoExplorer™ Software Evaluation

**Figure 10:**
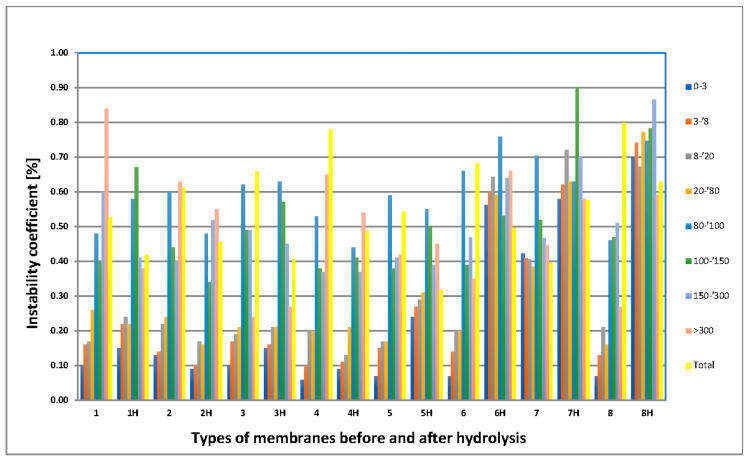
Influence of hydrolysis on the instability of membranes’ porosity in different types of membranes (horizontal axis) and size-classes of pores (groups of data).

**Figure 11:**
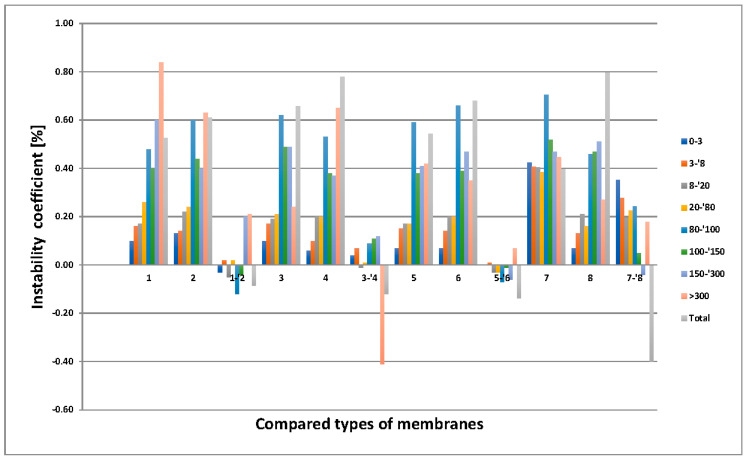
Influence of the using a solvent on the instability of membranes’ porosity in different types and size-classes of pores.

**Figure 12:**
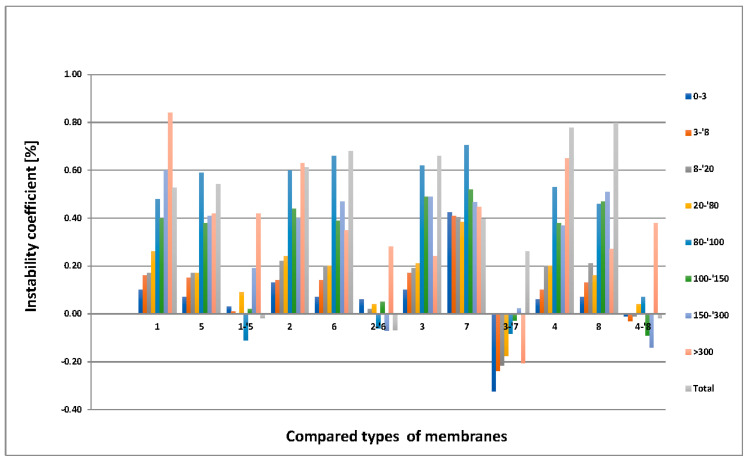
Influence of the addition of pore precursor on the instability of membranes’ porosity in different types and size-classes of pores.

The results of the statistics are shown in Figure 10, Figure 11 and Figure 12.

(a) Figure 10 presents the influence of hydrolysis on the instability of membranes’ porosity in different types and size-classes of pores. The data are presented in groups, each group containing the data corresponding to the eight size-classes of pores. On the other hand, the groups are presented in pairs corresponding to a given technology before and after hydrolysis.

Analysis of the corresponding data in pairs shows that, except for the type *i* = 7 of membrane, a *total instability* of porosity (yellow scores) decreased due to hydrolysis.

However, this effect in different size-classes of pores occurs with different intensities, as illustrated in Table 5. In the table + means increasing, ++ means high increasing, – means decreasing, and 0 means not observable influence of hydrolysis on the instability of porosity in the given size-class of pores and type of the membrane under examination.

It is remarkable that:

- The highest influence of hydrolysis on the reduction of the instability level occurs in the *j* = 5, 6, and 8 size-classes, while the lowest is in the *j =* 1 size-class of pores.
- The highest influence of membranes of hydrolysis on the reduction of the instability level can be observed in the *i =* 2 type of membrane, while the worse ones occurred the *I* = 6, 7, and 8 types of membranes.

(b) The results of the assessment of the influence of using a solvent on the instability of the membranes’ porosity are shown in Figure 11. The instability $d_{i,j,k}^{t}$ is presented in groups of scores corresponding to different size-classes of pores (*j*); the groups are arranged in four triplets, each triplet consisting of a pair of compared types of membranes (*i*) and their difference. The pairs that were taken into consideration are (1, 2), (3, 4), (5, 6), and (7, 8). The types of membranes not subjected to hydrolysis were compared. It is remarkable that the highest differences (the highest positive influence of using DMF as a solvent) occurred in the pairs of membranes (3, 4) and (7, 8), but in all compared membrane pairs the advantage of DMF over NMP in affecting the pore size is noticeable.

(c) The influence of the addition of pore precursor on the instability of membranes’ porosity in different types and size-classes of pores is illustrated in Figure 12. In this case, the following four pairs of types of membranes (1, 5), (2, 6), (3, 7), and (4, 8) were examined.

It is remarkable that the highest (positive) total influence occurs in the pair (3, 7) of membranes. However, the highest partial influence on the reduction of the instability occurs in the *7*th size-class of pores in the pairs (1, 5), (2, 6), and (4, 8) of membranes. Moreover, in allpairs of compared membranes negative partial influence in some classes of pores also occurred.

Referring to Figure 11 and Figure 12, the evaluation of SEM images using MeMoExplorer™ Software showed that the type of solvent used has an influence on the porosity of the membranes. Regardless of the pore class, the advantage of DMF over NMP was noticeable. The addition of PVP does not have such a significant effect on the porosity of the membranes, therefore, due to the fact that the deterioration of mechanical properties after hydrolysis was observed for membranes with PVP addition, PUR could be used as a pore precursor that would improve the useful life of membranes.

### 3.6. FT-IR Analysis

Comparative FT-IR spectra were performed to illustrate changes occurring in the membrane after the hydrolysis process. Fragments of the recorded spectra are listed in Table S1 in supplementary material. For all spectra, the peak fading is noticeable at the wavenumber around 1760–1730 and 1700–1630 cm^−1^. The 1760–1730 cm^−1^ peak is assigned to the C=O functional group derived from ester bonds, while 1700–1630 cm^−1^ can be attributed to the amine and carbamate groups. Although the FT-IR technique is not a method for quantification, it perfectly illustrates the changes that occur in the membrane after the hydrolysis process and proves that during hydrolysis, the ester or carbamate bonds contained in the PUR structure are broken down. The disappearance of the peaks for both functional groups leads to the conclusion that PUR hydrolysis takes place through several mechanisms simultaneously: the breakdown of ester bonds or carbamates. The advantage of one of the proposed mechanisms will be influenced by the arrangement of the PUR polymer chain in the membrane structure. Rather, it is a statistically dependent relationship on the position of the functional groups in the membrane.

## 4. Conclusions

The target of this study was to obtain a partly degradable PSF-PUR blend HFMs with constant PSF:PUR weight ratio. All received membranes were treated with the 1M NaOH solution and PUR was partially removed during the hydrolysis process, however PUR-2 with higher molar percentage of ester bonds was hydrolyzed with greater efficiency. A number of studies, experiments, and calculations show marked changes in morphology and membrane properties after the hydrolysis process. According to the assumptions of this study, hydrolysis process causes partial degradation of the membrane by hydrolysis of ester bonds contained in the PUR structure. Partial degradation of PUR in the membranes was confirmed by changes in the retention profiles of individual markers, increase in the UFC values after hydrolysis, structural changes imaged using SEM and evaluated using MeMoExplorer™ Software, and the disappearance of vibrations for the C=O, amine and carbamates functional groups. In this case PUR was used as a long-lived acting pore former and the degradation should have caused the increase of the porosity, UFC, and changes in retention without changes in the molecular weight cut-off. The authors hope that the obtained properties of the membranes have a chance to compensate for fouling (due to possibility of degradation) in biotechnological processes, especially in medical implants and the PUR is an alternative that can be used as a pore precursor instead of PVP or PEG. The higher UFC, porosity, and changes in retention may affect the duration of the membrane process in such cases when regeneration or replacement of membranes during the process is often impossible or very complicated. Potentially, such membranes may be used for macroencapsulation of biologically active agents, for example intended for a temporary implantation of several weeks.

## Figures and Tables

**Table 1:** Compositions of the membrane casting solutions.

Membrane	PUR	Solvent	PVP Addition
PSF-PUR-1	PUR 1	NMP	-
PSF-PUR-2		DMF	
PSF-PUR-3	PUR 2	NMP	
PSF-PUR-4		DMF	
PSF-PUR-5	PUR 1	NMP	50% of PSF weight
PSF-PUR-6		DMF	
PSF-PUR-7	PUR 2	NMP	
PSF-PUR-8		DMF	

**Table 2:** Size-classes of pores.

*j*	1	2	3	4	5	6	7	8
Size μm^2^	0–3	3–8	8–20	20–80	80–100	100–150	150–300	>300

**Table 3:** Ultrafiltration coefficients before and after the hydrolysis process.

Membrane	UFC$\;\left[\frac{cm^{3}}{\min\cdot m^{2}\cdot \mathrm{hPa}}\right]$	UFC_H_$\left[\frac{cm^{3}}{\min\cdot m^{2}\cdot \mathrm{hPa}}\right]$	$\frac{{\mathrm{UFC}}_{H}}{\mathrm{UFC}}$
PSF-PUR-1	0.0870 ± 0.0170	2.00 ± 0.02	23
PSF-PUR-2	1.14 ± 0.08	1.94 ± 0.17	1.7
PSF-PUR-3	2.76 ± 0.03	4.30 ± 0.34	1.6
PSF-PUR-4	0.0610 ± 0.0070	0.108 ± 0.002	1.8
PSF-PUR-5	0.0380 ± 0.0030	0.0830 ± 0.0350	2.2
PSF-PUR-6	0.141 ± 0.008	0.332 ± 0.097	2.4
PSF-PUR-7	0.929 ± 0.100	26.3 ± 0.2	28
PSF-PUR-8	0.125 ± 0.010	0.432 ± 0.049	3.5

**Table 4:** The results of PUR’s mass changes.

Membrane	Membrane’s Mass before Hydrolysis (g)	PUR’s Mass before Hydrolysis (g)	PUR’s Mass Loss after Hydrolysis (g)	PUR’s Mass Loss after Hydrolysis (%)
PSF-PUR-1	0.0890 ± 0.0026	0.0178	0.0082 ± 0.0003	46
PSF-PUR-2	0.100 ± 0.001	0.0200	0.0068 ±0.0023	34
PSF-PUR-3	0.0534 ± 0.0005	0.0107	0.010 ± 0.001	97
PSF-PUR-4	0.0946 ± 0.0027	0.0189	0.014 ± 0.001	75

**Table 5:** Results of hydrolysis influence on the instability of porosity.

*i* *j*	1	2	3	4	5	6	7	8
1	+	–	+	+	+	+	+	+
2	+	–	–	+	+	++	++	++
3	+	–	+	–	+	++	++	++
4	–	–	0	0	+	+	++	++
5	+	–	0	–	–	+	–	+
6	–	+	–	0	–	+	+	++
7	–	+	–	+	+	+	++	++
8	–	–	+	–	+	+	+	++

## Data Availability

The data presented in this study are available on request from the corresponding author. The data are not publicly available as the data is a part of PhD studies.

## Supplementary Materials

The following are available online at https://www.mdpi.com/2077-0375/11/1/51/s1, Table S1: Comparison of FT-IR spectra of membranes before and after hydrolysis. Figure S1: The cross-section and part of the cross-section of the PSF-PUR-1 membrane before (a) and after hydrolysis (b); Figure S2. The cross-section and part of the cross-section of the PSF-PUR-2 membrane before (a) and after hydrolysis (b); Figure S3. The cross-section and part of the cross-section of the PSF-PUR-3 membrane before (a) and after hydrolysis (b); Figure S4. The cross-section and part of the cross-section of the PSF-PUR-4 membrane before (a) and after hydrolysis (b); Figure S5. The cross-section and part of the cross-section of the PSF-PUR-5 membrane before (a) and after hydrolysis (b); Figure S6. The cross-section and part of the cross-section of the PSF-PUR-6 membrane before (a) and after hydrolysis (b); Figure S7. The cross-section and part of the cross-section of the PSF-PUR-7 membrane before (a) and after hydrolysis (b); Figure S8. The cross-section and part of the cross-section of the PSF-PUR-8 membrane before (a) and after hydrolysis (b).
