# Supplementary material for: Chemical Degradation of PSF-PUR Blend Hollow Fiber Membranes—Assessment of Changes in Properties and Morphology after Hydrolysis
Source: Membranes (Basel). 2021 Jan 12;11(1):51. doi: 10.3390/membranes11010051 (PMC7828234; doi:10.3390/membranes11010051)
Supplement: Supplementary file 1 [file membranes-11-00051-s001.pdf]

membranes

Supplementary Materials

Chemical Degradation of PSF-PUR Blend Hollow Fiber Membranes—Assessment of Changes in Properties and Morphology after Hydrolysis

Table S1. Comparison of FT-IR spectra of membranes before and after hydrolysis.

Membrane	Before hydrolysis	After hydrolysis
PSF-PUR-1	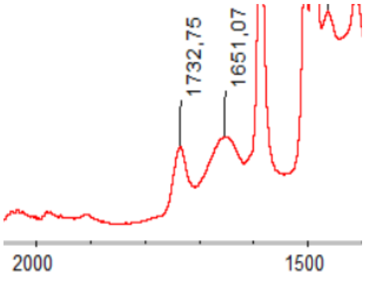	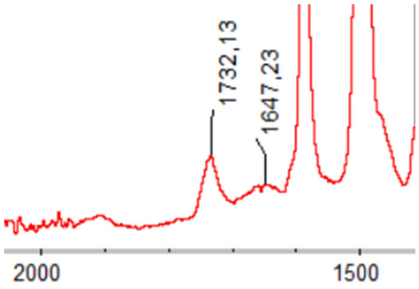
PSF-PUR-2	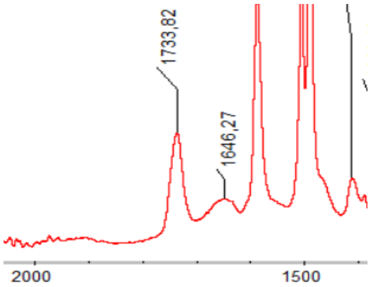	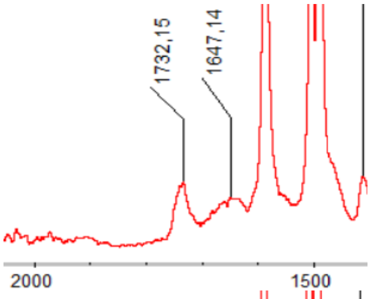
PSF-PUR-3	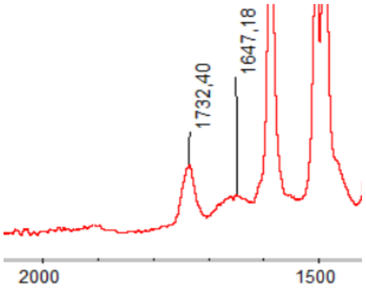	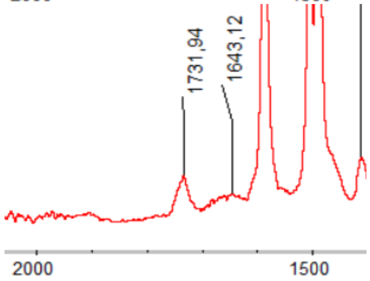
PSF-PUR-4	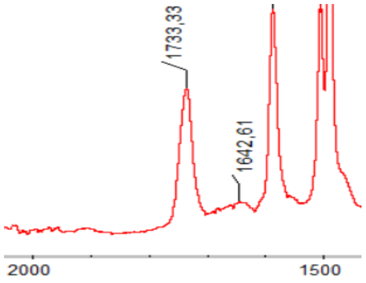	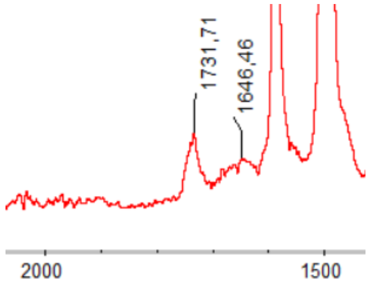

PSF-PUR-5

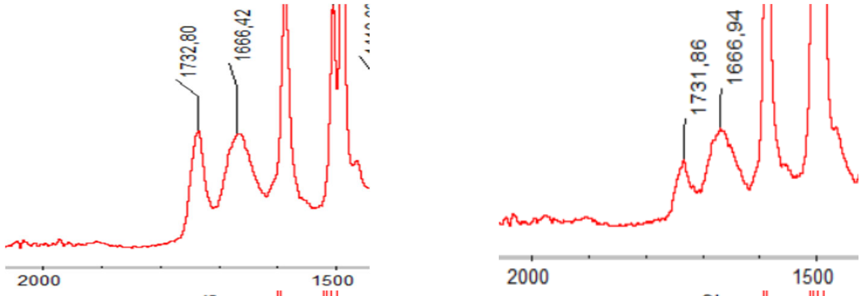

PSF-PUR-6

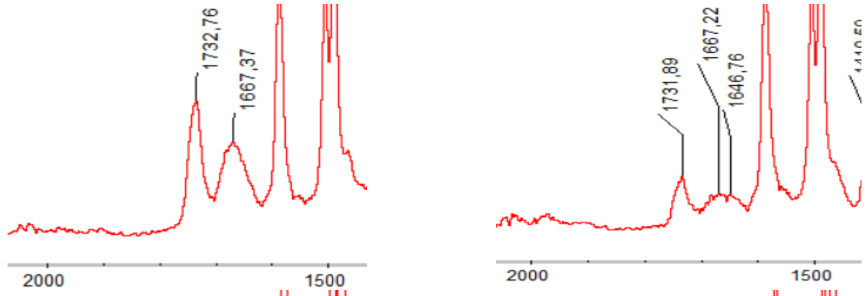

PSF-PUR-7

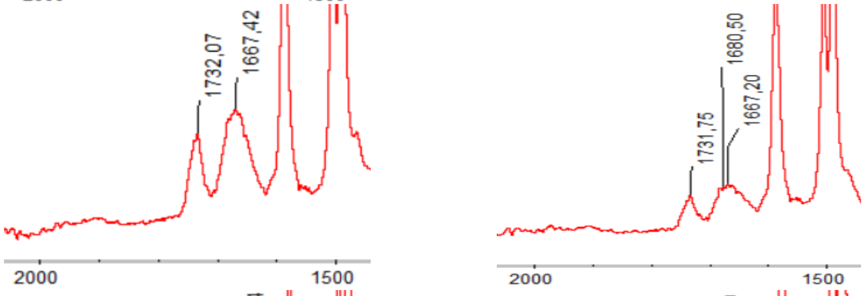

PSF-PUR-8

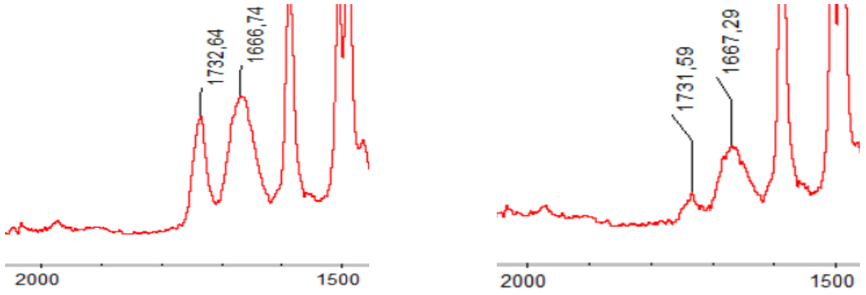

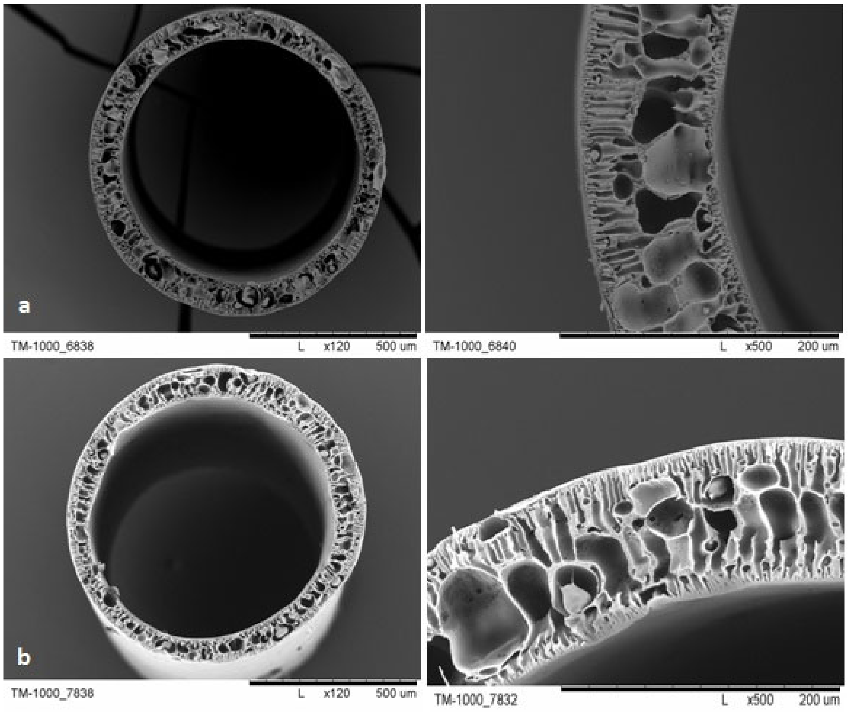

Figure S1. The cross-section and part of the cross-section of the PSF-PUR-1 membrane before (**a**) and after hydrolysis (**b**).

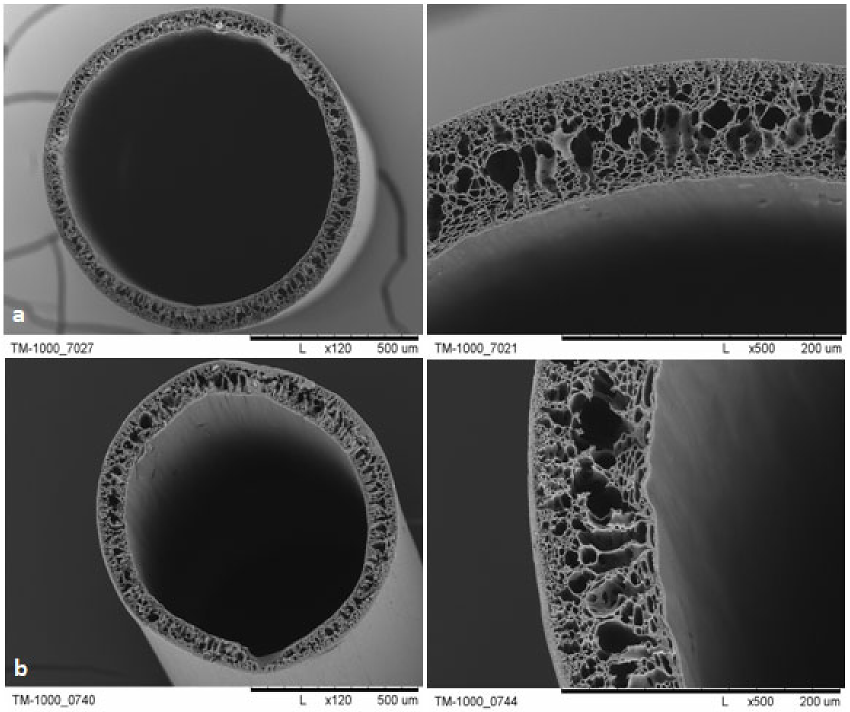

Figure S2. The cross-section and part of the cross-section of the PSF-PUR-2 membrane before (**a**) and after hydrolysis (**b**).

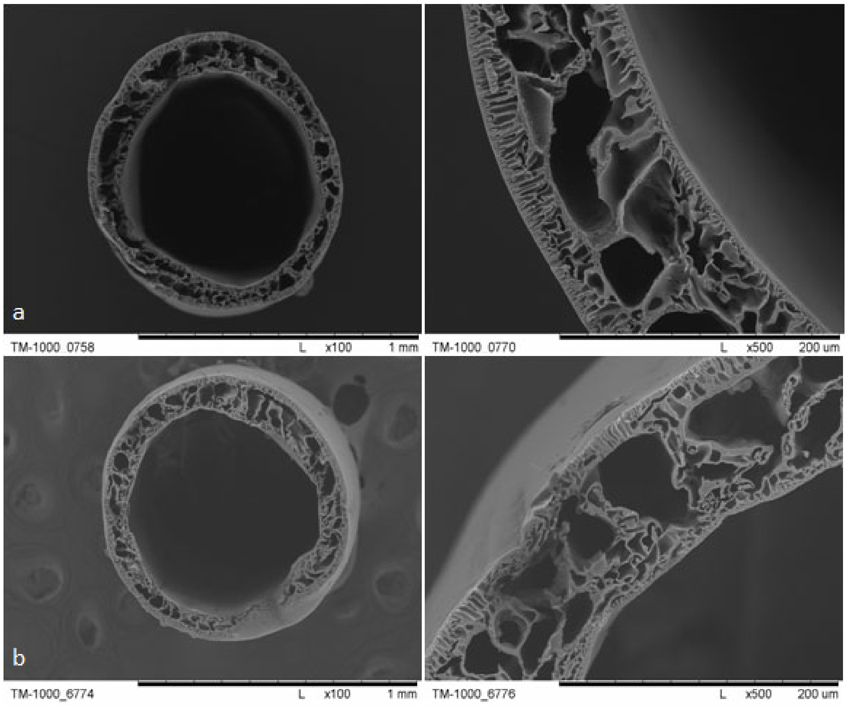

Figure S3. The cross-section and part of the cross-section of the PSF-PUR-3 membrane before (**a**) and after hydrolysis (**b**).

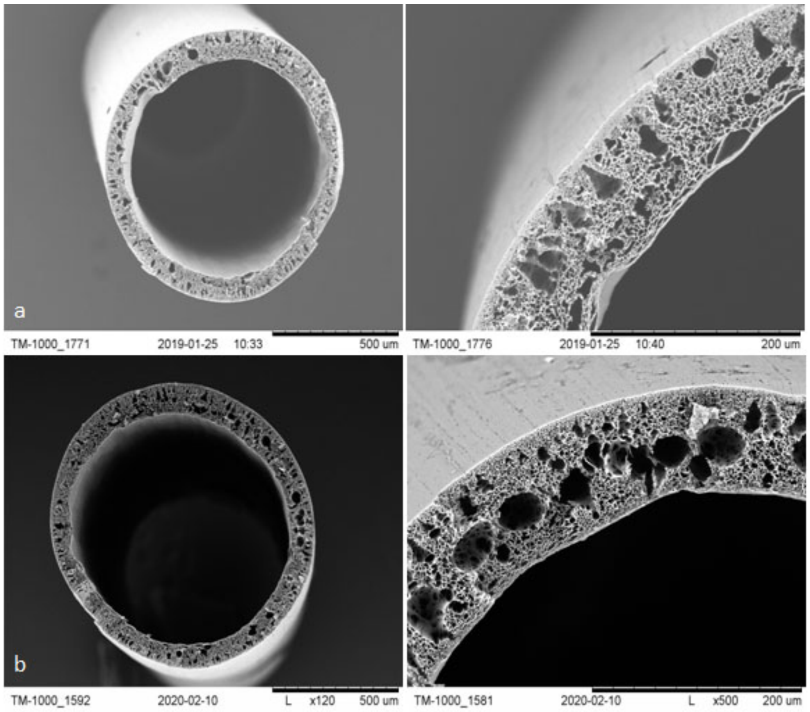

Figure S4. The cross-section and part of the cross-section of the PSF-PUR-4 membrane before (**a**) and after hydrolysis (**b**).

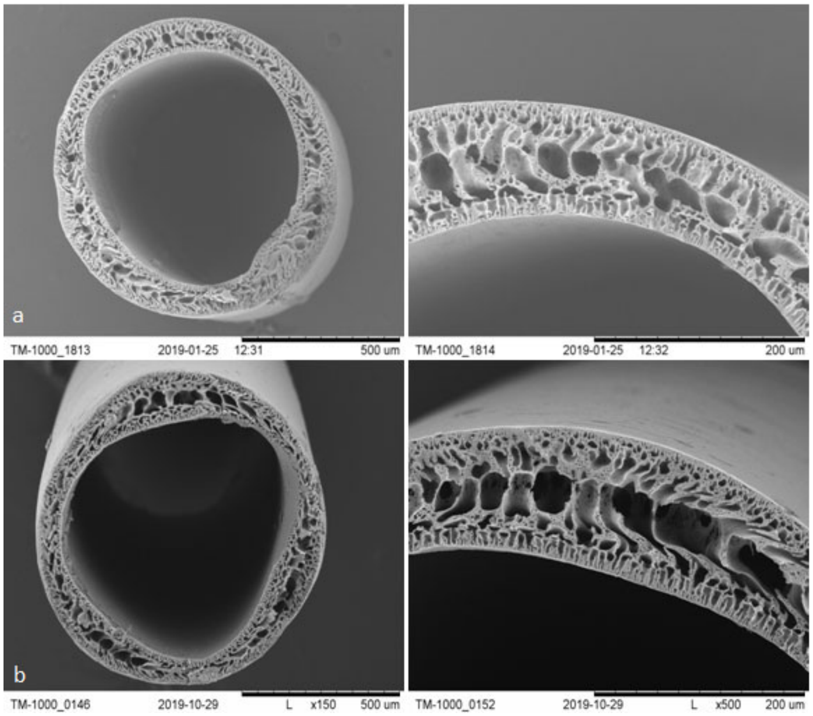

Figure S5. The cross-section and part of the cross-section of the PSF-PUR-5 membrane before (**a**) and after hydrolysis (**b**).

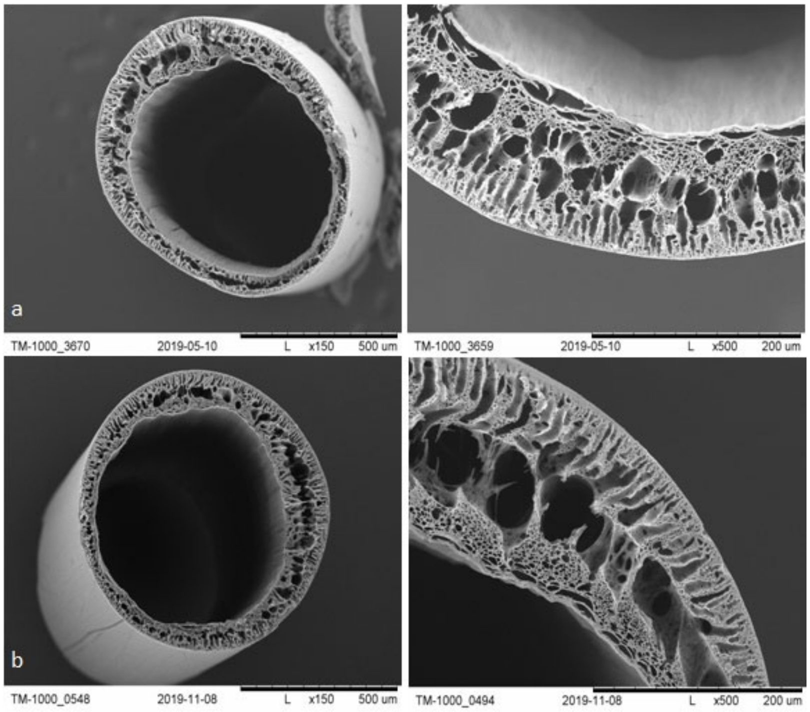

Figure S6. The cross-section and part of the cross-section of the PSF-PUR-6 membrane before (**a**) and after hydrolysis (**b**).

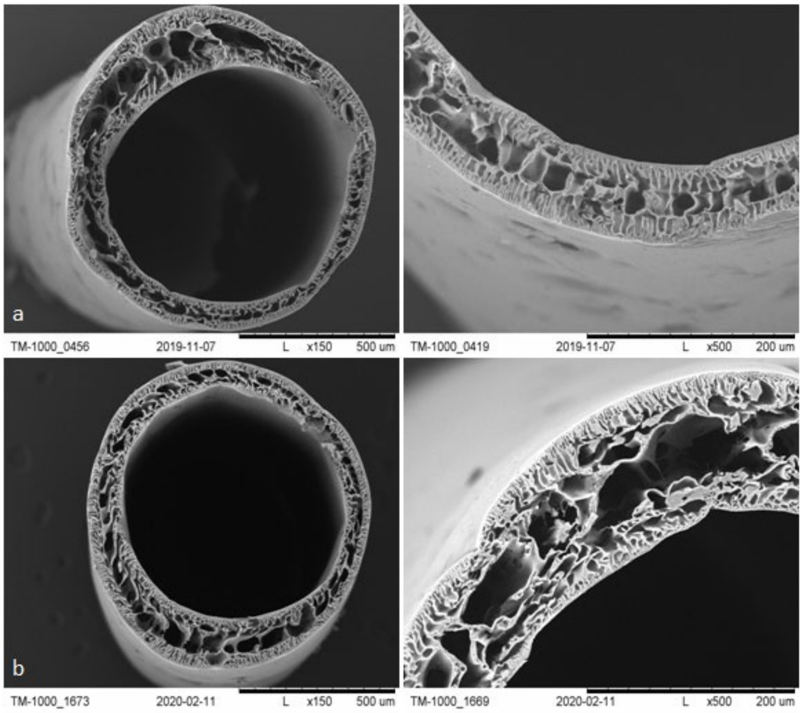

Figure S7. The cross-section and part of the cross-section of the PSF-PUR-7 membrane before (**a**) and after hydrolysis (**b**).

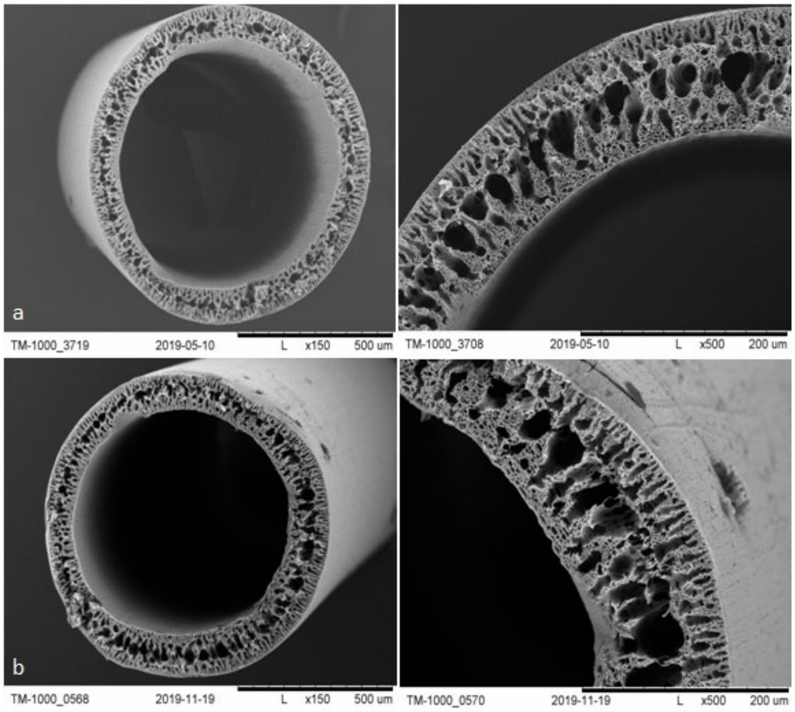

Figure S8. The cross-section and part of the cross-section of the PSF-PUR-8 membrane before (**a**) and after hydrolysis (**b**).
